# Health literacy among adolescents: a cluster analysis

**DOI:** 10.3389/fpubh.2026.1853107

**Published:** 2026-10-20

**Authors:** Solveig Veshovda, Kåre Rønn Richardsen, Marit Helen Andersen, Britt Elin Øiestad, Hedda Eik, Kirsti Riiser

**Affiliations:** 1 Department of Rehabilitation Science and Health Technology, Faculty of Health Sciences, Oslo Metropolitan University, Oslo, Norway; 2 Faculty of Health and Social Sciences, University of South-Eastern Norway, Drammen, Norway; 3 Department of Interdisciplinary Health Sciences, Faculty of Medicine, Institute of Health and Society, University of Oslo, Oslo, Norway; 4 Department of Transplantation Medicine, Oslo University Hospital, Oslo, Norway; 5 Department of Child and Adolescent Health Promotion Services, Norwegian Institute of Public Health, Levanger, Norway

**Keywords:** adolescent, cluster analysis, health behavior, health literacy, public health

## Abstract

**Background:**

Adolescents represent a critical target group for health literacy (HL) research, as this stage of life involves increasing independence and responsibility for personal health behaviors. The growing burden of health challenges among adolescents highlights the importance of HL, as it equips individuals to navigate such challenges and actively engage with their health. Despite growing recognition of HL as a critical determinant of health, research on adolescent HL remains limited. This study aimed to describe HL among adolescents aged 14–16 years and to identify and characterize HL cluster affiliations.

**Materials and methods:**

This cross-sectional study included adolescents aged 14–16 years across six schools in Southern Norway. Data were collected between March and September 2023 using a web-based questionnaire. The study employed the Health Literacy for School-Aged Children (HLSAC) instrument (range 10–40). HL clusters were identified based on the instrument’s five core HL components (theoretical knowledge, practical knowledge, individual critical thinking, self-awareness, and citizenship) using Ward’s method. We employed multiple multinomial regression analyses to examine factors associated with different cluster affiliations.

**Results:**

The total sample’s (*n* = 440) mean score for HL was 31.6. The analysis indicated four HL clusters, reflecting ordered levels of HL, from low (Cluster A) to high (Cluster D). Adolescents with higher academic ambitions and fathers with a higher educational level had a lower relative risk (RRR) of being affiliated with the lowest HL cluster. Males had a lower RRR than females of being affiliated with the second-lowest HL cluster compared to the highest HL cluster.

**Conclusion:**

This study used cluster analysis to examine HL among adolescents, identifying four HL clusters ranging from low to high. The findings reveal social influences on HL, such as academic ambition and parental education, and how health determinants might shape its distribution at the population level. These data-driven insights underscore the need for further research into health outcomes.

## Introduction

In recent years, policymakers have become more aware of the importance of enhancing the population’s health literacy (HL) to improve individuals’ ability to manage their health (1–3). Possessing HL emerges as crucial, enabling individuals to access, comprehend, and act upon health information effectively (4, 5). Adolescents represent a critical target group for HL research, as this stage of life involves increasing independence and responsibility for personal health behaviors, and evidence suggests that health behavior tracks from childhood into adulthood (6). Variation exists within the scientific community regarding the conceptual understanding and terminology of HL, resulting in diverse interpretations of the concept in health and medical research (7). In this study, HL is understood as the competence and motivation to obtain and construct health information, make sound health decisions, and change the factors that constitute one’s own health and that of others (8).

Globally, low HL is linked to poorer health outcomes and greater healthcare use (9–11). Among adolescents, limited HL poses significant challenges (4, 12–14). Promoting adolescent health, preventing disorders, and addressing issues early is essential during this formative stage, as cognitive, emotional, and biological development influence health behaviors (15–17). Previous studies indicate that girls generally demonstrate higher HL levels than boys, which may be linked to a greater interest in education and stronger academic performance (18, 19). Although awareness of HL in adolescents has grown over the past decade (20, 21), much of the research has assessed HL in relation to individual roles such as peers or caregivers (22).

The growing burden of health challenges among adolescents highlights the importance of HL, as it equips individuals with the skills, knowledge, and critical thinking to find, assess, and use health information and engage with healthcare systems more effectively. For instance, there has been a notable increase in pain issues among adolescents over the past decades, with persistent pain affecting 20 to 35% worldwide (23–25). The adverse effects of adolescent pain encompass various challenges and those afflicted often report a major impact on daily living (26), including less participation in leisure activities, more school absence, a restricted social life, and lower health-related quality of life (24, 27–30). Adolescents’ effective use of health information may support pain management and overall well-being. However, research on the associations between HL and pain, especially among adolescents, remains limited (31).

Adolescents are constantly exposed to health-related information from a wide range of sources, including peers, adults, social media, and commercial enterprises. It is recognised that HL depends on contextual factors, such as origin, environmental demands, and available resources, which can either limit or enhance an individual’s opportunities. For adolescents, these opportunities are often shaped by their socio-demographic background and surroundings, potentially creating disparities in access to health-promoting resources (21, 23, 32, 33). A key part of this context is the role of parents, as adolescents often rely on their parents’ knowledge and guidance in navigating health-related decisions. In line with this, previous research highlights parental education as a critical determinant of adolescent health outcomes, including HL (33, 34).

Despite growing recognition of HL as a critical determinant of health, research focusing on adolescents remains limited compared to studies on adults. Existing evidence links HL to health behaviors and outcomes in adolescents (35), but much of this research has centered on functional HL, such as basic reading and writing skills, leaving broader dimensions underexplored. Expanding the scope of HL research among adolescents is essential to develop evidence-based, targeted interventions that address their specific needs. Cluster analysis offers a valuable approach for identifying patterns within HL data, capturing variations in competencies and providing a more comprehensive understanding of adolescents’ HL. These patterns may differ across various characteristics, such as gender, educational ambition, pain, origin, and parental education. Thus, the primary objectives of this study were to describe health literacy (HL) among adolescents aged 14–16 years, to identify HL clusters, and to describe which factors are associated with different HL clusters.

## Materials and methods

### Study design and setting

This is a cross-sectional study including Norwegian adolescents (14–16 years of age) that was carried out across three different municipalities in southern Norway. All study procedures were approved by Sikt, the Norwegian Agency for Shared Services in Education and Research (reference no. 991780). The Strengthening the Reporting of Observational Studies in Epidemiology (STROBE) guidelines were followed to ensure the reporting quality of this study (see Additional File 1) (36).

### Recruitment of participants

The recruitment of study participants was conducted through sequential sampling with assistance from local school physiotherapists, ensuring that the researchers did not directly select participants. Six lower secondary schools were invited and selected according to geographical region, school size, and urban or rural location. The schools received written information about the study in advance of data collection, which was made available to adolescents and parents prior to the study. Participation in the study was voluntary, and informed consent was obtained from the study participants. Written consent from parents or caregivers of adolescents in lower secondary school was obtained prior to data collection. No participants with parental consent declined to participate.

### Data collection

The data collection was conducted between March and September 2023 via a web-based questionnaire, completed in the classroom during school hours. The data collection utilised a pseudo-code directly linked to the consent form signed by the parents, ensuring that the data were de-identified. The questionnaire was administered via the encrypted version of the web-based questionnaire,^1^ and data are stored in Services for Sensitive Data (TSD) administered by the University of Oslo (37). Representatives from the school health service (school physiotherapists and/or nurses) and the first author (SV) were present during all assessments and available for questions throughout data collection. All participants were thoroughly briefed on the questionnaire details before data collection to reduce misunderstandings and to encourage accurate responses. Prior to data collection, the questionnaire was pilot-tested by six adolescents (aged 14–16) who provided feedback on the visual design and written presentation. Based on their feedback, minor adjustments were made to the questionnaire layout and presentation, including correction of spelling errors and page breaks. The pilot also identified topics that some adolescents found challenging, informing researchers to be present and answer questions during data collection.

### Variables

The questionnaire used in the study was based on individual items specifically developed for this study, items published in previous studies, and previously validated instruments, as referenced in the upcoming variable descriptions.

#### Outcome variable

HL was measured with the Health Literacy for School-Aged Children (HLSAC) questionnaire (range 10–40) (8, 38), a generic 10-item one-factor questionnaire covering five core components of HL: theoretical knowledge (item 1 and 5), practical knowledge (item 4 and 7), individual critical thinking (item 3 and 9), self-awareness (item 8 and 10), citizenship (item 2 and 6), see Table 1 (8, 38). The components, representing broader competence areas, are interconnected but not strictly hierarchical. They reflect the progression of HL from basic health topic literacy to a deeper understanding, enabling recognition and use of potential within oneself, others, and the wider world (38). Each item was rated on a 4-point scale ranging from 1 (“not at all true”) to 4 (“absolutely true”). Originally, HLSAC was developed and tested among Finnish adolescents 13- and 15-years and is based on five theoretically defined HL components, while psychometric testing supported a one-factor 10-item instrument (38). Although confirmatory factor analysis (CFA) has been conducted on a sample of Norwegian adolescents aged 16 years and older, the instrument has not yet been validated among Norwegian adolescents aged 13–15 years, which corresponds to the age group for which the instrument was originally developed (8, 39). In the present sample, the internal consistency of the HLSAC total scale was good (Cronbach’s α = 0.87).

**Table 1:** The core HL components (8, 38).

The contextualization of HL	
Theoretical knowledge Item 1 and 5	*Theoretical knowledge* refers to an understanding of health-related concepts, facts, and factors that influence health. It encompasses foundational health knowledge and the ability to recall and recognize relevant information when considering health issues.
Practical knowledge Item 4 and 7	*Practical knowledge* refers to the ability to apply health-related knowledge and skills in specific situations. It is often shaped by personal experiences and contextual understanding. This component includes everyday health skills, such as finding health information, accessing relevant health services, and responding appropriately to health-related needs.
Individual critical thinking Item 3 and 9	*Individual critical thinking* refers to the ability to reflect on, evaluate, and interpret health information. It involves curiosity, a desire to understand health issues in greater depth, and the capacity to analyze information, assess its credibility, solve problems, and make informed judgments about health-related matters.
Self-awareness Item 8 and 10	*Self-awareness* refers to the ability to reflect on one’s own health, experiences, values, and behaviors. It involves understanding how personal thoughts, feelings, and motivations relate to health-related choices and actions. This component also includes the capacity to learn from experiences and apply this understanding to support health and well-being.
Citizenship Item 2 and 6	*Citizenship* refers to the ability to consider how individual actions influence the health and well-being of others and society. It involves awareness of personal rights and responsibilities, acting in a socially responsible manner, and recognizing factors that affect opportunities for health at both the individual and community level.

Component descriptions are condensed versions of the original HLSAC framework. HL, health literacy; HLSAC, Health Literacy for School-Aged Children.

#### Independent variables

The socio-demographic variables included age, gender, educational ambition, parental educational level. Age had two response categories: 14–15 years and 16 years. Gender was measured with three response options: “boy,” “girl,” and “prefer not to say”. Educational ambition was assessed by asking, “How much education do you think you will complete?” with four response categories: (1) “High school/vocational training”, (2) “Three years of higher education”, (3) “More than three years of higher education”, and (4) “Don’t know.” This variable was collapsed into three response categories for the analyses: “≤Vocational studies/high school”, “≥College/university”, and “Don’t know.” Adolescents were also asked about their parents’ highest educational attainment with four response options: “Elementary school”, “High school”, “College/university”, and “Don’t know.” In the analysis, this variable was collapsed into the following categories: “Elementary school/high school”, “College/university”, and “Don’t know.”

Persistent pain was measured using an item from a large Norwegian cohort study (Fit Futures), which has shown good concurrent validity and test–retest reliability in adolescents (40). Participants were asked: “Do you have persistent or recurring pain that has lasted for 3 months or more?”. The response options were “Yes” or “No”.

#### Descriptive variables

Origin was assessed through an open-ended response format asking participants to indicate the country in which they were born or the country in which their parents were born. The variable was further dichotomized into two groups: “Norwegian” and “Not Norwegian” (all countries other than Norway). Additionally, one question targeting adolescents’ overall health (41, 42) was included and phrased as follows: “Generally, how would you rate your health?”. The item was rated on a 5-point scale ranging from 1 (“Excellent”) to 5 (“Poor”). These responses were categorized into three categories for descriptive reasons: “Excellent/very good,” “Good/fair” and “Poor”. Lastly, we added a question adapted from a previous study (13), asking: “What sources of health information do you use if you have pain or injuries?”. Respondents could select up to three of 12 alternatives. In the analysis the sources were categorized into the following groups: digital sources, parents/family/friends, and health personnel.

### Statistical methods

#### Sample size

The sample size was determined based on plans to conduct cluster analysis and multiple regression analyses (43), aiming for approximately 10–15 individuals per degree of freedom in the model. This estimate accounted for independent variables with an additional 20% included to accommodate potential missing data. Following discussions within the project group, a chosen sample of *n* = 350 was determined to enhance the robustness and reliability of the findings, as well as to balance statistical power and feasibility (44). The sample size was also dependent upon participant availability during the data collection process.

#### Analysis

Continuous variables are described using mean and standard deviation (SD), while categorical variables are summarized with counts and percentages.

This study applied Ward’s Hierarchical Clustering method (45) to uncover groups of individuals who reported similar patterns in HL scores across the five core HL components: theoretical knowledge, practical knowledge, individual critical thinking, self-awareness, and citizenship, as operationalized by Paakkari and Paakkari (8) and Paakkari et al. (38) (Table 1). Ward’s method measures cluster adequacy by evaluating distances between cluster centroids (a measure of cohesion) and seeks to minimize the total within-cluster variance, resulting in different cluster solutions at various levels of the hierarchy (45). The core HL components were created by summing pairs of related items to form component scores.

To determine the optimal number of clusters, we used a combination of quantitative and qualitative considerations, including cluster size, within-cluster variability, between-cluster differentiation, dendrogram inspection, and interpretability of the resulting patterns (46). Theoretical considerations referred to the framework of HLSAC and findings in previous HL research that suggest a meaningful and interpretable number of subgroups. The dendrogram was examined through visual inspection to assess the merging process. Component scores and SD were used descriptively to assess cluster similarity and differentiation (45–47). For example, larger SD were interpreted as indicating greater within-cluster heterogeneity, whereas smaller SD indicated greater homogeneity. However, these measures were considered qualitatively alongside other criteria and were not used as distinct cut-off criteria when selecting the final cluster solution. The final clusters’ mean HL total scores and component scores are presented in a data-driven heatmap to illustrate HL patterns (Table 2).

**Table 2:** HL clusters, mean HLSAC scores, and descriptive background statistics for the total sample and clusters (*N* = 440).

HLSAC	Total	Cluster A	Cluster B	Cluster C	Cluster D
*N* (%)	440 (100)	70 (16)	135 (31)	129 (29)	106 (24)
HLSAC total score, Mean (SD)	31.6 (4.41)	25.3 (3.17)	29.3 (1.47)	33.1 (1.40)	37.1 (1.76)
Health literacy components, Sum Score, (SD)					
Theoretical knowledge		5.3 (0.98)	5.9 (0.58)	6.6 (0.79)	7.5 (0.57)
Practical knowledge		5.2 (0.96)	6.3 (0.79)	6.9 (0.81)	7.7 (0.44)
Critical thinking		5.0 (0.85)	5.5 (0.81)	6.1 (0.74)	7.2 (0.73)
Self-awareness		5.3 (0.98)	5.7 (0.70)	7.1 (0.71)	7.5 (0.77)
Citizenship		4.4 (1.01)	5.8 (0.58)	6.4 (0.87)	7.2 (0.68)

Variables	*N* (%)	*N* (%)	*N* (%)	*N* (%)	*N* (%)
Persistent pain ≥ 3 months	209 (48)	39 (56)	62 (46)	63 (49)	45 (43)
Gender					
Female	212 (48)	24 (35)	75 (56)	66 (51)	47 (44)
Male	217 (49)	42 (60)	57 (42)	63 (49)	55 (52)
Prefer not to say	10 (2)	3 (4)	3 (2)	–	4 (4)
Missing	1 (0)	1 (1)	–	–	–
Origin, Norwegian	405 (92)	63 (91)	130 (96)	117 (91)	95 (90)
Educational ambition					
≤High school/vocational studies	116 (26)	35 (50)	36 (27)	27 (21)	18 (17)
≥College/university	222 (50)	18 (26)	62 (46)	71 (55)	71(67)
Do not know	101 (23)	17 (24)	37 (27)	30 (23)	17 (16)
Missing	1 (0)	–	–	1 (1)	–
Health information source^a^					
Digital sources (internet, SoMe, ChatGPT)	311 (71)	51 (73)	86 (64)	97 (75)	77 (73)
Parents, family, friends	354 (80)	47 (67)	108 (80)	113 (88)	86 (81)
Health personnel (doctor, physiotherapist, nurse, psychologist, other)	297 (68)	36 (51)	88 (65)	96 (74)	77 (73)
Health (self-rated)					
Excellent/very good	194 (44)	28 (40)	47 (35)	55 (43)	64 (60)
Good/fair	206 (47)	35 (50)	72 (53)	63 (49)	36 (34)
Poor	33 (8)	6 (9)	13 (10)	9 (7)	5 (5)
Missing	7 (2)	1 (1)	3 (2)	2 (2)	1 (1)
Origin mother, Norwegian	362 (82)	56 (80)	118 (87)	104 (81)	84 (79)
Origin father, Norwegian	361 (82)	53 (76)	117 (87)	107 (83)	84 (79)
Educational level, mother					
≥College/university	229 (52)	25 (36)	57 (42)	74 (57)	73 (69)
≤Elementary/high school	104 (24)	21 (30)	33 (24)	29 (23)	21 (20)
Do not know	105 (24)	24 (34)	44 (33)	25 (19)	12 (11)
Missing	2 (0)	–	1 (1)	1 (1)	–
Educational level, father					
≥College/university	173 (39)	12 (17)	49 (36)	58 (45)	54 (51)
≤Elementary/high school	144 (33)	33 (47)	40 (30)	38 (29)	33 (31)
Do not know	122 (28)	25 (36)	46 (34)	32 (25)	19 (18)
Missing	1 (0)	–	–	1 (1)	–

Identified clusters and summed HLSAC scores per core HL components for each cluster (min 2–max 8) (HLSAC total score = 10–40) followed by descriptive background statistics for the total sample and the different clusters (*n* = 440). Scores marked in red indicate the lowest scores, orange indicates low scores, yellow indicates moderate scores, and green indicates higher scores.

Values are number (*N*) (%) if not otherwise indicated.

a Respondents could select up to three options from a total of 12 alternatives. The responses were further categorized into the following source groups: digital sources, parents/family/friends, and health personnel.

HL, health literacy; SD, standard deviation; HLSAC, Health Literacy for School-Aged Children; SoMe, social media.

Finally, a multinomial regression model with the identified HL clusters as the dependent variable was fitted to assess cluster affiliation, i.e., whether selected variables (persistent pain, gender, and socio-demographic variables) were associated with different clusters, with cluster D as the reference category (Table 3) (48). First, we screened candidate variables using univariable multinomial regression analyses and chi-square analyses. Candidate variables with *p* < 0.2 in both analyses, along with variables of known clinical importance, in this case persistent pain, were included as candidate variables in the multiple multinomial regression model (Additional File 2) (49, 50). The descriptive variables, including origin and parental origin, were not included as independent variables in the final multinomial analysis due to highly skewed distributions, making them unsuitable for further modeling (49). *p*-values of <0.05 were considered statistically significant. Participants with missing data (7% of cases) on the outcome measure were excluded from the analyses. All analyses were performed using the Stata statistical software system, version 17.0 (51).

**Table 3:** Adjusted RRRs for HL cluster membership from multivariable multinomial regression analysis (*n* = 440).

Factors/clusters	Cluster A		Cluster B		Cluster C		Cluster D
No. (%)	70 (16)		135 (31)		129 (24)		106 (29)
	RRR (CI)	*p* value	RRR (CI)	*p* value	RRR (CI)	*p* value	[ref]
Persistent pain ≥ 3 months							
Yes	1.60 (0.81–3.15)	0.18	1.27 (0.73–2.20)	0.39	1.27 (0.74–2.17)	0.38	[ref]
No	[ref]		[ref]		[ref]		[ref]
Gender							
Female	[ref]		[ref]		[ref]		[ref]
Male	0.88 (0.43–1.80)	0.73	0.49 (0.27–0.87)	0.01^*^	0.69 (0.39–1.20)	0.19	[ref]
Educational ambition							
≤High school/vocational studies	[ref]		[ref]		[ref]		[ref]
≥College/university	0.17 (0.07–0.41)	0.00*	0.41 (0.19–0.86)	0.02^*^	0.65 (0.31–1.38)	0.27	[ref]
Do not know	0.61 (0.24–1.57)	0.31	0.96 (0.41–2.25)	0.92	1.16 (0.48–2.79)	0.73	[ref]
Educational level, mother							
≤High/elementary school	[ref]		[ref]		[ref]		[ref]
≥College/university	0.51 (0.20–1.28)	0.15	0.50 (0.22–1.09)	0.08	0.68 (0.31–1.47)	0.33	[ref]
Do not know	2.87 (0.75–11.00)	0.12	2.01 (0.64–6.27)	0.22	1.12 (0.35–3.57)	0.84	[ref]
Educational level, father							
≤High/elementary school	[ref]		[ref]		[ref]		[ref]
≥College/university	0.37 (0.14–0.94)	0.03*	0.96 (0.46–2.00)	0.93	1.09 (0.54–2.21)	0.80	[ref]
Do not know	0.46 (0.14–1.55)	0.21	0.92 (0.34–2.51)	0.87	1.20 (0.45–3.20)	0.71	[ref]

Estimates of marginal probabilities (ranging from 0 to 1) and RRRs for adolescents belonging to one of the HL clusters derived from an adjusted multivariable multinomial regression model (*n* = 440). ^*^p < 0.05. RRR, relative risk ratios; CI, confidence interval; Ref., reference; HL, health literacy; *N*, number.

### Ethics

The study was reviewed by the Norwegian Regional Committees for Medical and Health Research Ethics (REK), which concluded that the project did not require formal ethical approval (reference no. 251776). The processing of personal data was approved by Sikt (reference no. 991780). Participation was voluntary, informed consent was obtained from all parents/caregivers, and all procedures were conducted in accordance with the Declaration of Helsinki (52).

## Results

### Descriptive data

Eligible participants included 940 adolescents from six schools, of whom 472 (50%) completed the survey. Response rates varied between schools, ranging from 42 to 66%. Of the 472 respondents, 440 had complete data on the outcome measure (HL) and were included in the final sample (see Participant Flow Chart, Additional File 3). The mean age of the respondents was 15.1 (SD 0.3) years, and 92% were of Norwegian origin. Nearly half of the sample reported having persistent pain (48%), while 44% reported excellent or very good perceived health. Overall, the total sample HLSAC mean score was 31.6 (SD 4.4) (Table 2). Additional File 4 displays the mean (SD) and median HL scores and the response distribution for each single HLSAC item. The mean scores for the individual HLSAC items ranged from 2.9 (item 2) to 3.4 (item 4). The response distribution for the HLSAC items indicated that 15% to 50% of the participants reported the highest level of HL across the 10 items.

### HL clusters

The cluster analysis indicated four HL clusters, with the number of adolescents in each cluster ranging from 70 to 135. Cluster A had the lowest, and cluster D had the highest core HL component scores (Table 2). In the following sections, we describe the core HL components of each cluster, including their socio-demographic characteristics.

Cluster A comprised 16% of the sample and this group included the adolescents with the lowest HLSAC total and HL component scores. Within this cluster, the theoretical knowledge component (5.3) and self-awareness (5.3) were higher than the other component scores. The lowest component score was observed for the citizenship component, involving the ability to take social responsibility and considering the impact of one’s actions on others (4.4). Within this cluster, 35% were girls, and 26% of the adolescents had an ambition of getting more than 3 years of higher education. Additionally, 36% of the mothers and 17% of the fathers had college or university education. More than half (56%) of the adolescents in this cluster reported having persistent pain.

Cluster B is the largest cluster, representing 31% of the sample. This cluster also represents adolescents with the second lowest HLSAC total and HL component scores. Within this cluster, the highest component scores were observed for the practical knowledge component (6.3). The HL component with the lowest score was individual critical thinking (5.5), entailing a lower tendency to critically evaluate health information or seek a deeper understanding of health issues. This HL component includes skills such as analyzing, problem solving, and assessing the validity of health information. Within this cluster, 55% were girls, and 42% of the mothers and 36% of the fathers were reported to have a college or university education. Moreover, 24% of the adolescents expressed an educational ambition to pursue more than 3 years of higher education. Within this cluster, 46% reported having persistent pain of 3 months or more.

Cluster C represented 29% of the sample, and this group included the adolescents with the second highest HLSAC total and HL component scores. Adolescents in this cluster had highest score on the self-awareness component (7.1) representing the ability to reflect on oneself and enabling the personal contextualization of health issues. The HL component with the lowest score was critical thinking (6.1). Within this cluster, 51% were girls, and 30% wanted to pursue higher education for 3 years or more. Additionally, 57% of the mothers and 45% of the fathers were reported to have a college or university education. In this group, 49% reported experiencing persistent pain lasting 3 months or longer.

Cluster D represented 24% of the sample. This group had the highest HLSAC total score, and the highest scores across all HL components. The highest HLSAC component score was observed for the practical knowledge component (7.7), representing procedural skills, enabling the application of theoretical knowledge in specific contexts. Rooted in personal experiences, it includes tacit, intuitive, or implicit understanding. In general, the adolescents in this cluster reported agreement with the HLSAC items, suggesting a high perceived level of HL. Their lowest scores were found in individual critical thinking and the citizenship component (7.2). Within this cluster, 67% reported that they wanted to pursue higher education for 3 years or more, 44% were girls, and a large percentage of adolescents had parents with higher education (51% of fathers and 69% of mothers). In this group, 43% reported experiencing persistent pain lasting 3 months or longer. See Figure 1 for a visualization of clusters A-D across the core HL components.

**Figure 1:**
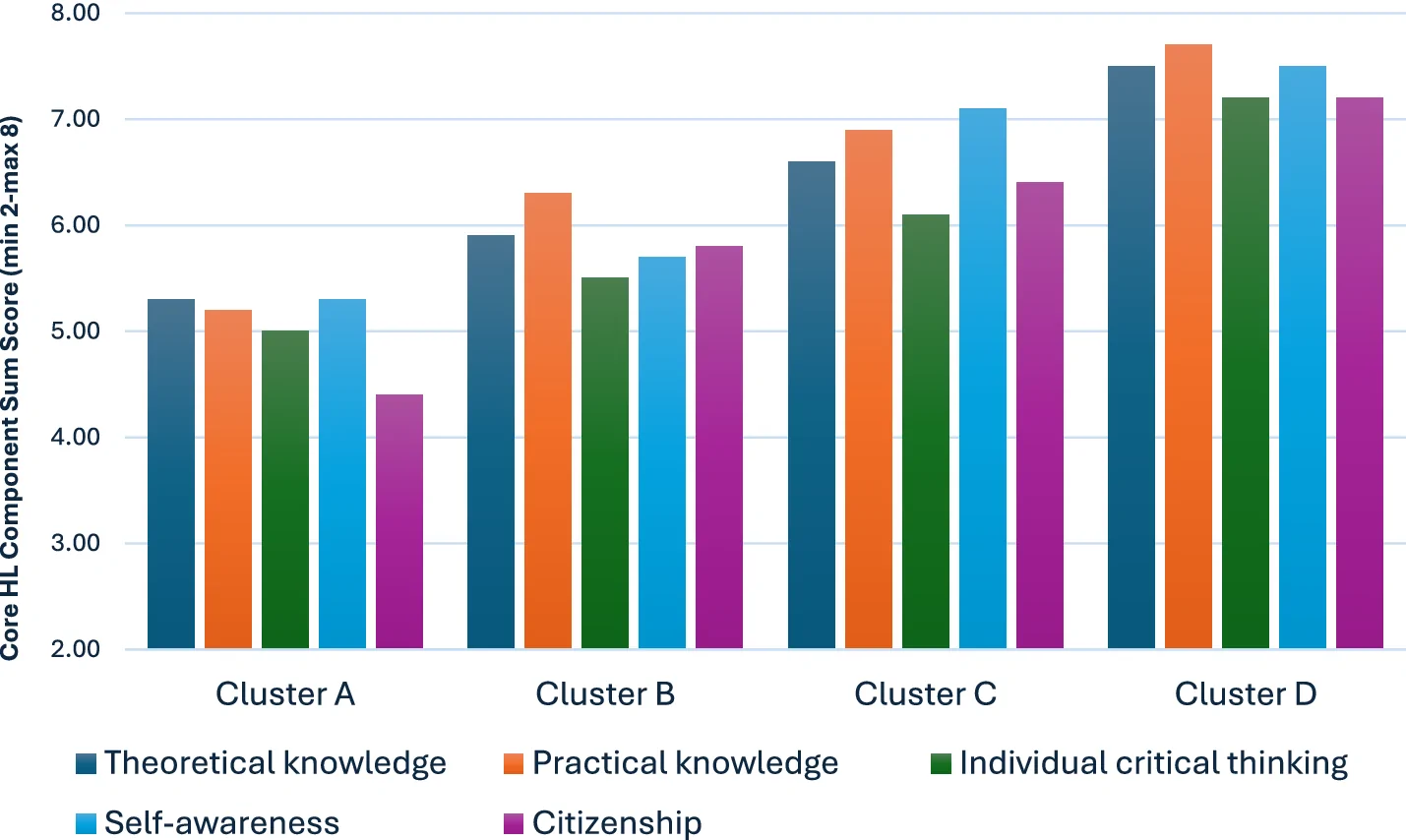
Distribution of the core HL components across four clusters (score range: min 2–max 8). Distribution of the core HL components (theoretical knowledge, practical knowledge, individual critical thinking, self-awareness, citizenship) across four clusters. Component scores range from min 2 to max 8. HL, health literacy.

### Cluster affiliation

Table 3 presents the relative risk (RRR) of affiliation with clusters A-C, contrasted with cluster D, given selected covariates. The analysis revealed that being male was associated with a 51% lower RRR of affiliation with cluster B contrasted with cluster D (RRR = 0.49, 95% CI: 0.27, 0.87, *p* = 0.01). Adolescents with an ambition to pursue a college or university education had an 83% lower RRR of affiliation with cluster A compared to cluster D (RRR = 0.17, 95% CI: 0.07, 0.41, *p* < 0.001). Similar results also apply for cluster B (RRR = 0.41, 95% CI: 0.19, 0.86, *p* = 0.02). Having a father with a high educational level was associated with 63% lower RRR of affiliation with cluster A compared to cluster D, (RRR = 0.37, 95% CI: 0.14, 0.94, *p* = 0.03). Further, the analysis indicated that adolescents with persistent pain did not have a significantly higher RRR of affiliation with cluster A compared to cluster D (RRR = 1.60, 95% CI: 0.81, 3.15, *p* = 0.18). Nor did having a mother with a higher educational level have a higher or lower RRR of affiliation with any of the clusters compared to cluster D.

## Discussion

The aim of this study was to describe HL among adolescents aged 14–16 years and to identify HL clusters and describe which factors are associated with different cluster affiliations. The overall mean HL score in our sample was comparable to or slightly higher than mean scores in previous studies (53, 54). This finding is expected, as our sample consists of adolescents at the upper end of the age range for which the HLSAC instrument was designed (38). In general, item 2 received the lowest mean score, which assesses the ability to give ideas on how to improve health in one’s immediate surroundings. Item 4 received the highest score, reflecting the adolescents’ ability to follow instructions given by health personnel. Four HL clusters were identified based on the five core HL components proposed by Paakkari and Paakkari (8) and Paakkari et al. (38). Together, these clusters reflected a graded pattern of HL, ranging from low (Cluster A) to high (Cluster D) levels across the summed score of the core HL components.

While the identified clusters showed some variation in their HL scores across the various components, the lowest component scores were observed in the citizenship and individual critical thinking components across various clusters. This might reflect how HL develops over time, with some components potentially requiring more cognitive effort or experience (38). However, while elements of progression exist, the components are not strictly hierarchical and may overlap. Lower scores on more advanced components may reflect variations in HL rather than inherent difficulty, underscoring the importance of considering individual and contextual factors in shaping adolescents’ HL.

Among the clusters, adolescents in cluster A scored lowest on the citizenship component, indicating that adolescents within this cluster might face challenges in addressing health-related issues (38). Without guidance, they might struggle to make informed health decisions and understand the broader impact of their actions on others. This highlights the need for societal efforts to support adolescents in developing a strong sense of social responsibility and awareness of how their actions affect others. Furthermore, citizenship also includes recognizing and addressing factors that affect both personal and collective health outcomes (15, 38).

Similarly, the individual critical thinking component, which entails skills such as validating health information and rationally understanding health issues, received the lowest or second-lowest scores across all clusters. Again, adolescents in cluster A scored particularly low on this component, but this was also evident in the other clusters (B, C, and D). Critical appraisal of health information is a fundamental aspect of HL, essential for fostering patient-centered care, empowerment, and informed decision-making (55, 56). The findings of this study highlight the need to strengthen appraisal competencies among adolescents to equip them for navigating complex healthcare systems and making informed health decisions. However, while research on adolescent critical HL is expanding, most school-based approaches have primarily focused on information appraisal, often overlooking broader dimensions of critical thinking, such as understanding social determinants of health, citizenship, and health-related actions (56–58). Recent studies emphasize the need for comprehensive assessments that capture these aspects, ensuring a deeper understanding of health determinants (56–58).

This study’s findings reveal that adolescents in clusters A and B had a notably low proportion of parents with higher education, for both mothers and fathers. This distribution is further supported by the multinomial analysis, which showed that having a father with a high educational level was associated with a lower RRR of belonging to cluster A compared to cluster D. Parental education likely plays an important role in shaping adolescents’ HL, potentially influencing their ability to critically assess and apply health information. These findings align with existing research, which consistently highlights parental education as a critical determinant of adolescent health outcomes, including HL (33, 34). Adolescents are especially dependent upon their parents or caregivers HL for access to material, financial, and social resources (e.g., health care) (16). Parents with higher education may facilitate access to health information and support effective communication, while lower education levels might limit adolescents’ exposure to health-promoting behaviors and resources. Given that HL develops within social and environmental contexts, adolescents with less exposure to adults with higher educational backgrounds may have fewer opportunities to engage in discussions that foster critical thinking and informed decision-making about health. This may also reflect differences in educational ambitions observed among the clusters identified in our study, which showed that adolescents aspiring to higher education were significantly less likely to belong to the lowest-scoring clusters (A and B) compared to the highest HL cluster (D).

In this study, there were no clear patterns in cluster affiliation based on gender; however, a higher distribution of boys was observed in the lowest HL cluster, while cluster B had a greater number of girls. The distribution was even in clusters C and D. Furthermore, the multinomial analysis revealed that being male was associated with a lower risk of belonging to cluster B compared to cluster D, the cluster with the highest HL. These findings might reflect potential gender differences in HL levels, highlighting variations in health-related engagement, communication patterns, or social expectations. Previous research shows that girls achieve higher HL levels than boys across various countries (18, 19). Following the literature, this may be explained by the fact that girls are more interested in education in general (59), succeed better in schools (60), and are often better educated in adulthood (61–63). Understanding these differences is essential for tailoring HL interventions to better support both boys and girls in developing health competencies.

Furthermore, in this study, we were interested in examining whether pain was associated with the level of HL. Nearly half of the adolescents in our sample reported experiencing persistent pain, which appears slightly higher than what has been reported in previous national population studies (26). A larger distribution of adolescents reporting pain was also found in the cluster with the lowest HL compared to the cluster with the highest HL. Research on the relationship between HL and pain in adolescents appears limited. While some previous reviews address related topics (24, 64–66), few directly examine HL as an associated factor. Previous research has reported small to large associations between HL and pain intensity in adults (67). Adults with higher HL tend to experience lower pain intensity, likely due to better pain management strategies (68). This study’s findings may reflect the multifaceted nature of adolescent pain (69), which is shaped by a complex interplay of individual and environmental factors (28, 70–74). Furthermore, relationships between pain and HL might be mediated by variables not directly captured in our analysis. There is a need for further investigation into how HL influences pain experiences and management strategies in adolescents (23–25). For instance, future studies could use qualitative designs to capture the complexity and dynamic nature of these interactions, as well as the broader socio-cultural and environmental contexts of adolescents’ lives.

### Strengths and limitations

This study is the first of its kind to classify adolescents into HL clusters based on their scores across the core HL components of HLSAC (8, 38). We chose to use cluster analysis in favor of the predefined cut-off values provided by the original authors (18), as cluster analysis allows for a more nuanced understanding of the data. This explorative and data-driven approach helped us uncover characteristics of different subgroups within our sample, avoiding a potential simplistic presentation of the phenomena of HL. While predefined cut-off values categorize HL into low (score 10–25), moderate (score 26–35), and high (score 36–40) (18), we believe that data-driven clusters can provide more meaningful insights and can better capture the complexities of HL. However, the clusters in this study show similarities with the predefined cut-offs, where cluster A is close to the threshold for low HL (18).

The selection of clusters was based on a combination of quantitative and qualitative considerations, including cluster size, within-cluster variability, between-cluster differentiation, dendrogram inspection, and interpretability of the resulting patterns (45). Solutions with two to five clusters were evaluated. For example, the two-cluster solution provided limited differentiation and broadly separated participants into lower and higher HL groups. The three-cluster solution offered greater differentiation; however, the distribution of participants across clusters was uneven and provided limited additional insight into patterns across the HLSAC components. Although a five-cluster solution also was explored, it largely reproduced the overall pattern observed in the four-cluster solution while further dividing the lowest HL group into very small clusters, resulting in limited interpretive value. In contrast, the four-cluster solution both captured the gradient in HL across the five HLSAC components and provided greater differentiation than the two- and three-cluster solutions, while maintaining adequate cluster sizes and avoiding the fragmentation observed in the five-cluster solution. It is important to acknowledge that this approach is sensitive to subjectivity, as it does not rely solely on statistical criteria to determine the optimal number of clusters (45, 75). There are no evidence-based guidelines defining what constitutes a relevant difference between the core HL component scores. However, three researchers (SV, KRR, and KR) collaboratively agreed on the most clinically meaningful cluster solution.

Several reviews have examined available instruments to measure HL in young people (19, 76, 77), but high-quality instruments remain scarce (19, 31), with few being both comprehensive and suitable for large-scale population surveys (76). The HLSAC instrument’s strength lies in its attempt to capture the multifaceted nature of HL (38). However, the instrument has not been validated in Norwegian for the age group 13–15 years (39), and there are no reference studies available for Norwegian adolescents. Although the instrument demonstrated good internal consistency in the current sample, these are important factors to consider when interpreting the results and applying them to broader contexts. Furthermore, although HLSAC is based on five theoretical components, it was psychometrically established as a one-factor instrument (38). Consequently, the cluster findings identified in the present study should be interpreted as reflecting theoretically informed components of HL rather than empirically established subscales.

A strength of this study is the wide geographic distribution of the sample. However, it is important to highlight that not all eligible participants had parental consent, leading to lower participation rates in some schools. This affected the overall sample size and may have limited the generalizability of the findings to the broader adolescent population (78). In addition, school-level clustering introduces potential similarities among participants, which were not accounted for in the analyses. This should be considered when interpreting the findings. The study relied on self-reported measures, which may have introduced reporting bias and affected the accuracy of some responses, particularly for background and sociodemographic variables. However, when HL is conceptualized as individuals’ perceived competencies, it can only be assessed through self-report (79).

## Conclusion

This study used cluster analysis to examine HL among adolescents, identifying four HL clusters ranging from low to high. The findings reveal social influences on HL, such as academic ambition and parental education, and emphasize how health determinants might shape its distribution at the population level. These data-driven insights underscore the need for further research into adolescent health outcomes.

## Data Availability

The datasets presented in this article are not readily available because the datasets generated and/or analyzed during the current study are not publicly available due to additional ongoing analyses and the potential for further studies to be published based on this data material but are available from the corresponding author on reasonable request. Requests to access the datasets should be directed to Solveig Veshovda, solvei@oslomet.no.

## Footnotes

1
www.nettskjema.no
