# Supplementary material for: Health literacy among adolescents: a cluster analysis
Source: Front Public Health. 2026 Oct 20;14:1853107. doi: 10.3389/fpubh.2026.1853107 (PMC13727571; doi:10.3389/fpubh.2026.1853107)
Supplement: Supplementary file 1 [file Supplementary_file_1.docx]

Additional File 1. STROBE Statement—checklist of items that should be included in reports of observational studies

|  | Item No. | Recommendation |  | Relevant text from manuscript |
| --- | --- | --- | --- | --- |
| **Title and abstract** | 1 | (*a*) Indicate the study’s design with a commonly used term in the title or the abstract |  | Health literacy among adolescents: a cluster analysis. |
|  |  | (*b*) Provide in the abstract an informative and balanced summary of what was done and what was found |  | Abstract including background, material and methods, results, conclusions and keywords. |
| Introduction | | | |  |
| Background/rationale | 2 | Explain the scientific background and rationale for the investigation being reported |  | Introduction chapter leading to the study’s objectives. |
| Objectives | 3 | State specific objectives, including any prespecified hypotheses |  | The primary objectives of this study were to describe health literacy (HL) among adolescents aged 14–16 years, to identify HL clusters, and to describe which factors are associated with different HL clusters. |
| Methods | | | |  |
| Study design | 4 | Present key elements of study design early in the paper |  | Key elements of the study design (cross-sectional study) are presented in the Abstract and described in detail in the Study Design and Setting subsection of the Methods section. |
| Setting | 5 | Describe the setting, locations, and relevant dates, including periods of recruitment, exposure, follow-up, and data collection |  | Details regarding the study setting, participant recruitment, timing of data collection, and study procedures are provided in the Recruitment and Data Collection subsections of the Methods section. |
| Participants | 6 | (*a*) *Cross-sectional study*—Give the eligibility criteria, and the sources and methods of selection of participants |  | Eligibility criteria and methods of participant selection are described in the Study Design and Setting and Recruitment of Participants subsections. |
|  |  | (*b*) *Cohort study*—For matched studies, give matching criteria and number of exposed and unexposed  *Case-control study*—For matched studies, give matching criteria and the number of controls per case |  | N/A |
| Variables | 7 | Clearly define all outcomes, exposures, predictors, potential confounders, and effect modifiers. Give diagnostic criteria, if applicable |  | Outcome variables, independent variables, and descriptive variables are clearly defined in the Variables section of the Methods chapter. Variables are described under separate subheadings, including definitions and measurement procedures. Diagnostic criteria N/A. |
| Data sources/ measurement | 8* | For each variable of interest, give sources of data and details of methods of assessment (measurement). Describe comparability of assessment methods if there is more than one group |  | Sources of data and methods of assessment for all variables are described in the Data Collection and Variables sections of the Methods chapter. Outcome variables, independent variables, and descriptive variables were assessed using validated self-report questionnaires and study-specific survey items. Assessment methods were identical for all participants. |
| Bias | 9 | Describe any efforts to address potential sources of bias |  | Efforts to minimize potential sources of bias are described throughout the Methods and Discussion sections. School-level clustering, and potential reporting bias and other study limitations are discussed in the Strengths and Limitations section. |
| Study size | 10 | Explain how the study size was arrived at |  | The study size is described in the Sample Size subsection of the Methods section. |

Continued on next page

| Quantitative variables | 11 | Explain how quantitative variables were handled in the analyses. If applicable, describe which groupings were chosen and why |  | Handling of quantitative variables is described in the Statistical Methods and Analysis subsections of the Methods section. Details regarding variable categorization and analytical procedures are provided in the Statistical Methods section. |
| --- | --- | --- | --- | --- |
| Statistical methods | 12 | (*a*) Describe all statistical methods, including those used to control for confounding |  | All statistical methods are described in the Statistical Methods and Analysis subsections of the Methods section. Descriptive, cluster, and multinomial regression analyses are reported, including the variables entered into the statistical models. |
|  |  | (*b*) Describe any methods used to examine subgroups and interactions |  | N/A |
|  |  | (*c*) Explain how missing data were addressed |  | Participants with missing data (7% of cases) on the outcome measure were excluded from the analyses. |
|  |  | (*d*) *Cohort study*—If applicable, explain how loss to follow-up was addressed  *Case-control study*—If applicable, explain how matching of cases and controls was addressed  *Cross-sectional study*—If applicable, describe analytical methods taking account of sampling strategy |  | The sampling strategy is described in the Study Design and Setting section. No analytical adjustments for sampling strategy were applied. Potential school-level clustering is discussed as a limitation. |
|  |  | (*e*) Describe any sensitivity analyses |  | N/A |
| Results | | | | |
| Participants | 13* | (a) Report numbers of individuals at each stage of study—eg numbers potentially eligible, examined for eligibility, confirmed eligible, included in the study, completing follow-up, and analysed |  | The number of participants at each stage of the study, including recruitment, participation, and inclusion in the analyses, is reported in the Results section. A participant Flow Chart is provided as an Additional File. |
|  |  | (b) Give reasons for non-participation at each stage |  | See additional file, Flow Chart. |
|  |  | (c) Consider use of a flow diagram |  | See additional file, Flow Chart. |
| Descriptive data | 14* | (a) Give characteristics of study participants (eg demographic, clinical, social) and information on exposures and potential confounders |  | Characteristics of the study participants, including sociodemographic variables, are reported in the Results section. Participant characteristics are presented in Table 2, which provides an overview of the study sample and relevant exposure variables. |
|  |  | (b) Indicate number of participants with missing data for each variable of interest |  | Information on missing data is reported in the Results section, Descriptive Data. |
|  |  | (c) *Cohort study*—Summarise follow-up time (eg, average and total amount) |  | N/A |
| Outcome data | 15* | *Cohort study*—Report numbers of outcome events or summary measures over time |  | N/A |
|  |  | *Case-control study—*Report numbers in each exposure category, or summary measures of exposure |  | N/A |
|  |  | *Cross-sectional study—*Report numbers of outcome events or summary measures |  | Summary measures for the outcome variable (health literacy) are presented in the Results section. |
| Main results | 16 | (*a*) Give unadjusted estimates and, if applicable, confounder-adjusted estimates and their precision (eg, 95% confidence interval). Make clear which confounders were adjusted for and why they were included |  | N/A. Statistical methods are described in the Statistical Methods and Analysis sections. Variables included in the univariable and multivariable multinomial regression analyses were selected based on predefined selection criteria, as described in the paragraph on multinomial regression. |
|  |  | (*b*) Report category boundaries when continuous variables were categorized |  | N/A |
|  |  | (*c*) If relevant, consider translating estimates of relative risk into absolute risk for a meaningful time period |  | N/A |

Continued on next page

| Other analyses | 17 | Report other analyses done—eg analyses of subgroups and interactions, and sensitivity analyses |  | N/A |
| --- | --- | --- | --- | --- |
| Discussion | | | | |
| Key results | 18 | Summarise key results with reference to study objectives |  | Key results are summarised at the beginning of the Discussion section, with explicit reference to the study objectives and research questions. |
| Limitations | 19 | Discuss limitations of the study, taking into account sources of potential bias or imprecision. Discuss both direction and magnitude of any potential bias |  | Limitations are discussed in the Strengths and Limitations section. |
| Interpretation | 20 | Give a cautious overall interpretation of results considering objectives, limitations, multiplicity of analyses, results from similar studies, and other relevant evidence |  | An overall interpretation of the findings is provided throughout the Discussion section. |
| Generalisability | 21 | Discuss the generalisability (external validity) of the study results |  | The generalizability of the findings is discussed in the Strengths and Limitations section. |
| Other information | |  | | |
| Funding | 22 | Give the source of funding and the role of the funders for the present study and, if applicable, for the original study on which the present article is based |  | The source of funding and the role of the funders are reported in the Funding and Declarations sections at the end of the manuscript. |

*Give information separately for cases and controls in case-control studies and, if applicable, for exposed and unexposed groups in cohort and cross-sectional studies.

**Note:** An Explanation and Elaboration article discusses each checklist item and gives methodological background and published examples of transparent reporting. The STROBE checklist is best used in conjunction with this article (freely available on the Web sites of PLoS Medicine at http://www.plosmedicine.org/, Annals of Internal Medicine at http://www.annals.org/, and Epidemiology at http://www.epidem.com/). Information on the STROBE Initiative is available at www.strobe-statement.org.

| Additional File 2. RRR for HL cluster membership from univariate multinomial regression analysis (n=440). | | | | | | | |  |
| --- | --- | --- | --- | --- | --- | --- | --- | --- |
| **Factors/clusters** | **Cluster A** | | **Cluster B** | | **Cluster C** | | **Cluster D** | **Chi square** |
| No. (%) | 70 (16) |  | 135 (31) |  | 129 (29) |  | 106 (24) | ***P* value** |
|  | **RRR (CI)** | ***P* value** | **RRR (CI)** | ***P* value** | **RRR (CI)** | ***P* value** | [ref] |  |
| **Persistent pain ≥ 3 months** |  |  |  |  |  |  |  |  |
| Yes | 1.70 (0.93-3.14) | 0.09* | 1.15 (0.69-1.92) | 0.59 | 1.29 (0.77-2.17) | 0.33 | [ref] | 0.36 |
| No | [ref] |  | [ref] |  | [ref] |  | [ref] |  |
| **Gender** |  |  |  |  |  |  |  | 0.05* |
| Female | [ref] |  | [ref] |  | [ref] |  | [ref] |  |
| Male | 1.50 (0.79-2.82) | 0.21 | 0.65 (0.39-1.09) | 0.10* | 0.82 (0.48-1.37) | 0.44 | [ref] |  |
| **Educational ambition** |  |  |  |  |  |  |  |  |
| ≤ High school/vocational studies | [ref] |  | [ref] |  | [ref] |  | [ref] | 0.00* |
| ≥ College/university | 0.13 (0.06-0.28) | 0.00* | 0.43 (0.22-0.85) | 0.01* | 0.67 (0.34-1.32) | 0.24 | [ref] |  |
| Don't know | 0.51 (0.21-1.24) | 0.14* | 1.09 (0.49-2.44) | 0.84 | 1.18 (0.51-2.73) | 0.71 | [ref] |  |
| **Educational level, mother** |  |  |  |  |  |  |  |  |
| ≤ High/elementary school | [ref] |  | [ref] |  | [ref] |  | [ref] | 0.00* |
| ≥ College/university | 0.34 (0.16-0.73) | 0.01* | 0.50 (0.26-0.95) | 0.03* | 0.73 (0.38-1.40) | 0.35 | [ref] |  |
| Don’t know | 2.00 (0.80-5.01) | 0.14* | 2.33 (1.01-5.41) | 0.05* | 1.51 (0.62-3.67) | 0.36 | [ref] |  |
| **Educational level, father** |  |  |  |  |  |  |  |  |
| ≤ High/elementary school | [ref] |  | [ref] |  | [ref] |  | [ref] | 0.00* |
| ≥ College/university | 0.22 (0.10-0.49) | 0.00* | 0.75 (0.41-1.37) | 0.35 | 0.93 (0.51-1.69) | 0.82 | [ref] |  |
| Don’t know | 1.31 (0.61-2.83) | 0.48 | 1.99 (0.99-4.05) | 0.06* | 1.46 (0.70-3.05) | 0.31 | [ref] |  |
| **Origin** |  |  |  |  |  |  |  |  |
| Norwegian | 0.21 (0.43-3.46) | 0.71 | 3.01 (1.01-8.95) | 0.05* | 1.13 (0.48-2.67) | 0.78 | [ref] | 0.20 |
| Not Norwegian | [ref] |  | [ref] |  | [ref] |  | [ref] |  |
| **Origin, mother** |  |  |  |  |  |  |  |  |
| Norwegian | 1.05 (0.49-2.21) | 0.90 | 1.82 (0.91-3.63) | 0.09* | 1.09 (0.57-2.07) | 0.79 | [ref] | 0.31 |
| Not Norwegian | [ref] |  | [ref] |  | [ref] |  | [ref] |  |
| **Origin, father** |  |  |  |  |  |  |  |  |
| Norwegian | 0.81 (0.40-1.68) | 0.58 | 1.80 (0.90-3.60) | 0.10* | 1.27 (0.66-2.46) | 0.47 | [ref] | 0.29 |
| Not Norwegian | [ref] |  | [ref] |  | [ref] |  | [ref] |  |
| Estimates of marginal probabilities (ranging from 0 to 1) and RRR for adolescents belonging to one of the HL clusters derived from chi-square and univariate multinomial regression model (n = 440). *p < .20. Abbreviations: Ref. = reference, HL = health literacy, RRR = relative risk ratio, CI = confidence interval. | | | | | | | |  |

**Participants invited**

n = 940

**Provided parental consent**

n = 531

**Not present at school during data collection**

n = 59

**Completed questionnaire**

n = 472

**Excluded from final analyses**

Missing data n = 32

**Included in final analyses**

n = 440

Additional File 3. Study participant Flow Chart. Abbreviations: n = number

| Additional File 4. Adolescents mean (SD) and median HL scores and the response distribution for each HLSAC item (1-4), (N=440). | | | | | | | | |
| --- | --- | --- | --- | --- | --- | --- | --- | --- |
| **HLSAC- components** | **Item** | **Mean** | **SD** | **Median** | **N (%)** | | | |
|  |  |  |  |  | Not At All True | Not Completely true | Somewhat True | Absolutely True |
| Theoretical knowledge | Item 1. Having good information regarding health  Item 5. Ability to give examples of things that promote health | 3.1  3.3 | .58  .62 | 3  3 | 6 (1)  2 (1) | 41 (9)  34 (8) | 302 (69)  232 (53) | 91 (21)  172 (39) |
| Practical knowledge | Item 7. Ability to find health-related information that is easy to understand  Item 4. Ability to follow the instructions given by doctors and nurses | 3.2  3.4 | .63  .66 | 3  4 | 1 (0)  4 (1) | 46 (11)  32 (7) | 237 (54)  181 (41) | 156 (36)  223 (51) |
| Individual critical thinking | Item 9. Ability to decide if health-related information is right or wrong  Item 3. Ability to compare health-related information from different sources | 3.0  3.0 | .64  .68 | 3  3 | 4 (1)  6 (1) | 87 (20)  72 (16) | 268 (61)  253 (58) | 81 (18)  109 (25) |
| Self-awareness | Item 10. Ability to justify one’s own choices regarding health  Item 8. Ability to judge how one’s own behaviour affects one’s health | 3.2  3.3 | .68  .65 | 3  3 | 7 (2)  2 (0) | 48 (11)  43 (10) | 242 (55)  216 (49) | 143 (33)  179 (41) |
| Citizenship | Item 6. Ability to judge how one’s own actions affect the surrounding natural environment  Item 2. Ability to give ideas on how to improve health in one’s immediate surroundings | 3.2  2.9 | .70  .65 | 3  3 | 6 (1)  10 (2) | 60 (14)  82 (19) | 227 (52)  280 (64) | 147 (33)  68 (16) |
| Abbreviations: SD = standard deviation, HL = health literacy, HLSAC = Health literacy for school-aged children questionnaire, N = number. | | | | | | | | |
